# News and Events of the Week

**Published:** 1911-04-22

**Authors:** 


### News and Events of the Week.

The Governments of Belgium and Germany have come to an arrangement with regard to the notification of cases of rabies in the frontier districts of the two countries.

Professor S. H. C. Martin, M.D., F.R.S., has been elected Dean of the Faculty of Medicine of London University for the remainder of the period 1910-12.

Dr. Charles Henry Bennett, M.D., of College House, Hammersmith, W., and of Bridge Road, Hammersmith, who died on February 16 last, has left estate of the gross value of £93,396 13s. 7d.

The City of London Year Book (5s. net., Messrs. W. H. and L. Collingridge, 148 Aldersgate Street, E.C.) for 1911 has just been issued. It contains a large amount of valuable and interesting reading regarding everything that has any connection with City life and work.

A committee has been formed at Montpellier, France, with the object of erecting a monument to Rabelais, doctor and professor. The execution of this monument has been confided to the sculptor Jacques Villeneuve. Subscriptions should be sent to the general treasurer, M. Taicheire, pharmacist, at Montpellier, 20 Rue Nationale.

The Società Italiana di Patologia, at a meeting held in 1910 at Modena, decided to invite pathologists to an international reunion at Turin in 1911 on the occasion of the festivities in connection with the Jubilee of Italian Independence. Meetings will be held in the Institute of Pathological Anatomy of the University of Turin from October 2 to October 5, 1911. The bureau of the International Congress is at the Institute of General Pathology, corso Raffaello 30, Turin, to which all desirous of information should address their communications.

The New Zealand Government has intimated that it will contribute the sum of £2,000 towards the expenses of the visit of the British Association for the Advancement of Science to Australia in 1914.

It has been decided that students in the State of South Carolina who fail in their examinations will be examined in order to determine whether or not they are suffering from bookworm infection, on the ground that the failure may be due to the disease as often as to mental inefficiency.

This week's issue of *Mayfair*—the leading London society journal—contains a coloured cartoon of Sir William Whitla, M.D., LL.D., Professor of Materia Medica in the University of Belfast, together with an interesting biographical sketch of this distinguished Irish physician.

The eighth annual Medical Study Tour organised by L'Enseignement Medicomutuel International will start on August 12 next and end on August 30. Denmark, Norway, and Sweden will be visited. Full details may be had on application to the Central Bureau, 12 Rue François-Millet, Paris, XVIme.

The use of a common drinking-cup or receptacle for drinking water in any public place or in any public institution, hotel, theatre, factory, public hall, or public school, or in any railroad station or ferry house in the city of New York or the furnishing of such common drinking-cup or receptacle for use in any such place, is now prohibited by law as the result of a bacteriological examination of various cups used in common showed the necessity for drastic action.

The fifth international congress of thalassotherapy will be held in Kolberg, Germany, from June 5 to 8.

An association entitled the "Lunacy Reform League" has been formed in Melbourne. Some abusive statements, such as that patients are "fed like dogs out of a cauldron," at such and such an asylum are met by the point-blank denial of the Inspector-General, who points out the improvements effected in the past two or three years. Dr. Jones further suggests that if the Lunacy Reform League would make clear what it wants, its suggestions would be carefully and calmly considered, the Department being always open to adopt any really practical reform within the limits of available income.

